# Mid‐Infrared Photoacoustic Stimulation of Neurons through Vibrational Excitation in Polydimethylsiloxane

**DOI:** 10.1002/advs.202405677

**Published:** 2024-07-12

**Authors:** Zhiyi Du, Mingsheng Li, Guo Chen, Maijie Xiang, Danchen Jia, Ji‐Xin Cheng, Chen Yang

**Affiliations:** ^1^ Department of Chemistry Boston University Boston MA 02215 USA; ^2^ Department of Electrical and Computer Engineering Boston University Boston MA 02215 USA; ^3^ Division of Materials Science and Engineering Boston University Boston MA 02215 USA; ^4^ Department of Biomedical Engineering Boston University Boston MA 02215 USA

**Keywords:** mid‐infrared, neural stimulation, photoacoustic

## Abstract

Photoacoustic (PA) emitters are emerging ultrasound sources offering high spatial resolution and ease of miniaturization. Thus far, PA emitters rely on electronic transitions of absorbers embedded in an expansion matrix such as polydimethylsiloxane (PDMS). Here, it is shown that mid‐infrared vibrational excitation of C─H bonds in a transparent PDMS film can lead to efficient mid‐infrared photoacoustic conversion (MIPA). MIPA shows 37.5 times more efficient than the commonly used PA emitters based on carbon nanotubes embedded in PDMS. Successful neural stimulation through MIPA both in a wide field with a size up to a 100 µm radius and in single‐cell precision is achieved. Owing to the low heat conductivity of PDMS, less than a 0.5 °C temperature increase is found on the surface of a PDMS film during successful neural stimulation, suggesting a non‐thermal mechanism. MIPA emitters allow repetitive wide‐field neural stimulation, opening up opportunities for high‐throughput screening of mechano‐sensitive ion channels and regulators.

## Graphical abstract

Photoacoustic emitters are emerging ultrasound sources offering high spatial resolution and ease of miniaturization. This work shows mid‐infrared vibrational excitation of C─H bonds in transparent polydimethylsiloxane films leads to highly efficient photoacoustic conversion. Neural stimulation with tunable precision from single cell to 200 µm diameter is achieved. Mid‐infrared photoacoustic stimulation has a potential in high‐throughput screening of neuron regulators.

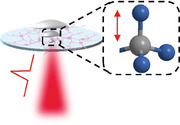

## 1 Introduction

Ultrasound, a mechanical wave with the frequency between 20 kHz and 1 GHz, has been applied in a variety of biomedical fields, such as non‐contact imaging,^[^ ^1^ ^]^ lithotripsy,^[^ ^2^ ^]^ tumor ablation,^[^ ^3^ ^]^ and neural modulation,^[^ ^4^ ^]^ owing to its high penetration in tissues. A piezoelectric transducer is usually used to efficiently generate the narrow band ultrasound with tunable pressures through the piezoelectric effect. However, the bulky size of the transducer and poor spatial resolution at the millimeter level make it challenging to integrate the transducer with widely used measurement modalities, including whole‐cell patch‐clamp electrophysiology or optical imaging.^[^ ^5^ ^]^ Alternatively, the photoacoustic (PA) effect, discovered by Bell in 1880, can generate broad‐band ultrasound under pulsed laser irradiation and subsequent thermal expansion of the material. The PA effect improves the spatial resolution of the ultrasound by confining the ultrasound in the illumination area.^[^ ^6^ ^]^ The photoacoustic effect of endogenous agents has been widely used in deep tissue imaging and has been shown to provide high‐resolution images at the depth of a few millimeters in soft tissues.^[^ ^7^ ^]^ Furthermore, compared to piezoelectric ultrasound transducers, photoacoustic transducers can be easily fabricated and miniaturized. The broad‐band ultrasound at the MPa level provided by photoacoustic emitters has enabled applications including ultrasound imaging,^[^ ^8^ ^]^ small particle manipulation,^[^ ^9^ ^]^ and neural modulation.^[^ ^10^ ^]^

Two basic components are required in a PA emitter: an optical absorber for photothermal generation and an elastomer for thermal expansion. In most cases, the absorbers are embedded in a matrix of elastomers such as polydimethylsiloxane (PDMS). Various materials have been explored as absorbers, including metals,^[^ ^8^, ^11^ ^]^ carbon materials,^[^ ^9^, ^12^ ^]^ and perovskites^[^ ^8a^ ^]^ for their strong absorption at the electronic transitions in the visible or near‐infrared (NIR) window. Despite strong optical absorption, the thermal conduction between absorbers and elastomers limits the conversion efficiency.^[^ ^13^ ^]^ In addition, the strong optical absorption in the visible window greatly reduces the transparency, making it difficult to collect optical signals in the same window with the presence of the PA emitter.

To address these limitations, we explore a new concept of highly efficient photoacoustic generation via direct bond‐selective excitation of the elastomer. Compared to electronic transitions in currently used absorbers, molecular vibrational transitions in the elastomer molecules are typically resonant with mid‐infrared light, resulting in high transparency in visible range. Significantly, although the vibrational transition cross section is much smaller than the electronic transition, the overall absorption remains large due to the high concentration of C─H bonds in these elastomers, for example, 80 mol kg^−1^ in the PDMS.

Vibrational transitions have been used in bond‐selective photoacoustic spectroscopy and microscopy.^[^ ^14^ ^]^ For example, detecting the C─O bond vibrational excitation can be used to sense CO gas at ppb levels,^[^ ^15^ ^]^ while detecting C─H bond vibrational excitation can help image the lipid distribution in living cells.^[^ ^7a^ ^]^ Despite these advances, the use of bond‐selective photoacoustic signals for cell modulation has not been explored.

Herein, we report a mid‐infrared photoacoustic (MIPA) emitter through vibrational excitation of C─H bonds in a transparent PDMS film. We show that a 25‐µm thick PDMS film generates 1.7 MPa PA pressure on the surface upon excitation by a 0.2 µJ MIR laser pulse at the wavelength of 3.38 µm for C─H stretching vibrational resonance. Furthermore, we systematically studied the relationship between Young's modulus and PA generation of the PDMS. By optimizing the Young's modulus of the PDMS film, a 26% enhancement of PA generation has been achieved. The optimized PA conversion efficiency was found to be 37.5 times higher than the commonly used opaque carbon materials‐based PA emitter. By surface modification, we demonstrate the good biocompatibility of PDMS films with cortical neurons.

The high photoacoustic conversion, transparency, and good biocompatibility enable the integration of the MIPA emitter with fluorescence microscopy to modulate and monitor neuron activities simultaneously. We demonstrate two stimulation modalities: a wide‐field stimulation in a field of view with a 100 µm radius and single‐cell stimulation through a focused mode. Furthermore, owing to the low thermal diffusion coefficient and the short irradiation duration, a temperature increases of less than 0.5 °C on the PDMS surface was found during the MIPA process. This result confirms that MIPA stimulation is safe and non‐thermal, unlike photothermal neural stimulation,^[^ ^16^ ^]^ or infrared neural stimulation.^[^ ^17^ ^]^ Given the fact that C─H bonds exist ubiquitously in organic materials, our MIPA design offers a new strategy to study cellular activities upon simultaneous stimulation and imaging.

## 2 Results

### 2.1 Exciting the Vibrational Transition of C─H Bonds in a PDMS Film Produces a Strong PA Signal

**Figure 1:**
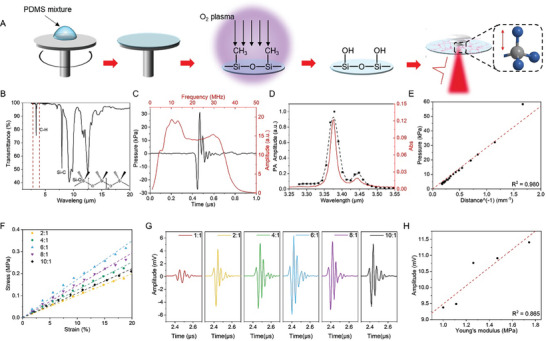
PDMS: The bond selective photoacoustic (PA) emitter. A) Schematic of the fabrication of the hydrophilic PDMS film as a transparent and biocompatible MIPA emitter. B) FTIR spectrum of the PDMS film. Inset: the chemical structure of PDMS. C) The photoacoustic waveform in the time domain (Black) and frequency domain (Red) of a 25‐µm thick PDMS film. D) The normalized PA amplitude and the absorption spectrum of a 25‐µm thick PDMS film in the mid‐infrared region. Black dots: photoacoustic pressure measured by a 25 MHz ultrasound transducer. Black dashed curve: B‐spline fitted curve for the PA spectrum. Red curve: Absorbance. E) Initial peak‐to‐peak photoacoustic pressures measured at different distances from the PA film. Black dots: experimental data. Red dashed line: the fitted curve. F) Stretching stress versus stretching strain of PDMS films prepared with different base‐to‐agent ratios. Dashed lines: linear fitted curves. G) Representative waveforms measured from different samples in Figure 1F. H) The PA amplitude measured and obtained from Figure 1G as a function of Young's modulus. Laser condition for this figure: 3.38 µm wavelength, 10 ns pulse width, 0.2 µJ pulse energy.

To validate whether mid‐infrared (MIR) excitation of a vibrational transition can effectively generate PA signals, we fabricated biocompatible PDMS films as shown in **Figure** **1**A. The pre‐mixed PDMS was spin‐coated on a clean CaF_2_ substrate followed by a 10 min curing at 120 °C. Uniform and flexible PDMS films were obtained with the thickness ranging from 4 to 90 µm, controlled by the spin‐coating speed (Figures S1 and S2, Supporting Information).

We measured the absorption of PDMS by an FTIR spectrometer. As shown in Figure 1B, the PDMS film showed an absorption peak at the wavelength of 3.38 µm, corresponding to the stretching vibrational transition of C─H bonds. The extinction coefficient of PDMS at 3.38 µm is 0.037 corresponding to an absorption depth of 7.3 µm.^[^ ^18^ ^]^ This strong absorption is attributed to abundant methyl groups in the PDMS Matrix (Figure 1B inset). According to Beer's law, the intensity of light at a certain depth of PDMS can be described by:

(1) $$I\left(d\right)=I_{0}\;\;e^{-\alpha d}=I_{0}\;e^{-4\pi d˛/\lambda }$$

where *I(d)* is the light intensity at the depth *d (m)*, *I_0_* is the incident light intensity, *α* is the absorption coefficient (m^−1^), *κ* is the optical extinction coefficient, and *λ* is the wavelength of the light (m).^[^ ^19^ ^]^ At 25 µm depth, the light intensity is attenuated to 3%. To minimize the light leakage, all PDMS films used were thicker than 25 µm, unless otherwise specified.

To demonstrate vibrational photoacoustic generation, we utilized a 10‐ns mid‐infrared OPO laser tunable from 2.56 to 3.75 µm. We tuned the MIR laser to 3.38 µm, focused it on the bottom of a PDMS film, and placed a 40‐µm diameter needle hydrophone 600 µm above the PDMS film. The recorded PA signal shows a high initial peak‐to‐peak pressure of 58 kPa with a central frequency of 10 MHz and a ‐3 dB bandwidth of 30 MHz (Figure 1C). This broadband frequency spectrum is consistent with a typical PA spectrum.^[^ ^20^ ^]^ We also quantitatively confirmed the linear relationship between laser fluence and generated PA pressure by tuning the laser energy delivered to PDMS from 0 to 0.2 µJ (Figure S3, Supporting Information). The PA spectrum of the PDMS film matched well with the fourier trasform infrared (FTIR) spectrum (Figure 1D), confirming that the generated PA signal originates from the C─H vibrational transition in PDMS.

Under the focused MIR condition, the diameter of the photoacoustic field was characterized as 10 µm (Figure S4, Supporting Information). Given the fact that the diameter of the ultrasound detector foci is 75 µm, much bigger than 10 µm, the illumination diameter is determined to be 10 µm.^[^ ^21^ ^]^ It is much smaller than the photoacoustic wavelength of 150 µm at the frequency of 10 MHz. Therefore, PDMS under MIR illumination can be treated as a point source. Theoretically, the PA pressure from a point source follows the equation of *P(r)* ∝ *1/r*, where *P* is the PA pressure and *r* is the distance between the detection position and the bottom surface of the PDMS film, which is illuminated by MIR. Figure 1E shows the experimentally measured PA pressure *P* as a function of 1/*r*. The fitting indicates that *P* is linear with 1/r. Together, our result demonstrates that PDMS can act as an efficient MIPA emitter.

To compare the performance of MIPA with other PA transducers, we prepared a carbon nanotube (CNT) embedded PDMS (CNT‐PDMS) film according to a previously reported method (Supporting information).^[^ ^22^ ^]^ The fabricated CNT‐PDMS film was measured to be 100 µm thick with 99% absorption of 532 nm light (Figure S5A, Supporting Information). The absorption depth for the CNT‐PDMS film at 532 nm is calculated to be 20 µm, twice as large as that of the PDMS film at 3.38 µm, indicative of its lower absorption at 532 nm. The concentration of CNTs in the PDMS matrix is typically less than 10% w/w. Further increasing the concentration of CNTs in PDMS could cause severe aggregation of CNTs and uneven film formation. As shown in Figure S5B and C (Supporting Information), under the same laser energy density, the CNT‐PDMS film produced a PA signal with a peak‐to‐peak amplitude of 0.6 mV. In comparison, the 25 µm thick PDMS film with a 6:1 base‐to‐agent ratio produced a MIPA signal with a peak‐to‐peak amplitude of 12.25 mV (Figure 1G). The result shows a 37.5 times higher PA conversion efficiency in PDMS compared to CNT‐PDMS. Furthermore, as shown in Figure S5C and D (Supporting Information), the PA amplitude generated by the CNT‐PDMS film is linearly dependent on the pulse energy, indicating no damage to the film. The PA waveform generated by CNT‐PDMS films showed a lower central frequency compared to the MIPA from PDMS, which can be attributed to the larger absorption depth.^[^ ^23^ ^]^ We reason that in common PA transducers composed of absorbers embedded in a matrix of elastomers, the light energy is absorbed by the absorber and converted to heat. The generated heat partially transfers to the surrounding elastomers to induce thermal expansion and to produce ultrasound.^[^ ^13^ ^]^ In MIPA, PDMS directly absorbs light energy and goes through thermal expansion, resulting in a higher PA conversion efficiency.

### 2.2 Optimization of Photoacoustic Efficiency and Biocompatibility of PDMS Films

The PA theory suggests the generated PA pressure is proportional to the bulk modulus of the material *Κ*, a measure of the mechanical properties of the material.^[^ ^6b^ ^]^ Specifically, the PA pressure can be described by:

(2) $$P\;=\;\Gamma \left(K\right)\mu_{a}F$$

where Γ is the Grüneisen parameter of the elastomer as a function of bulk modulus of the materials *Κ* (Pa), µ_*a*_ is the absorption coefficient (m^−1^), and *F* is the laser fluence (J/m^2^).^[^ ^6^, ^24^ ^]^ According to the definition of the Grüneisen parameter and using Maxwell relations, the Grüneisen parameter can be reconfigured into Γ = β*K*/*C*ρ, where *β* is the thermal expansion coefficient (K^−1^), *C* is the specific heat (J kg^−1^ K^−1^), and *ρ* is the density of PA materials (kg m^−3^).^[^ ^8^, ^23^ ^]^ Given the fact that the bulk modulus *K* is related to the Young's modulus *Y* (Pa) with the following relationship: K=Y/(3(1−2/μ)), where *µ* is the Poisson ratio of PDMS (*µ* ≈0.45^[^ ^25^ ^]^). Together, the pressure is proportional to Young's modulus of PDMS.

One way to tune the elasticity of PDMS is to adjust the base‐to‐curing agent ratio.^[^ ^26^ ^]^ We prepared PDMS films using different base‐to‐curing agent ratios, from 10:1 to 2:1, and measured the stress and strain of the samples. The slope of the stress‐strain curve for each sample indicates Young's modulus. As shown in Figure 1F, the PDMS sample with a 6:1 base‐to‐curing agent ratio shows the highest Young's modulus with a value of 1.745 MPa. We then measured the PA pressure of these films under the same laser conditions with a 25 MHz ultrasound transducer (Figure 1G). As expected, the PDMS film with the base‐to‐curing agent ratio of 6:1 showed the highest PA amplitude. Comparing to the PDMS film with a 10:1 base‐to‐curing agent ratio, the PDMS film with a base‐to‐curing agent ratio of 6:1 shows a 26% increase in the PA amplitude. Furthermore, as shown in Figure 1H, the PA signal amplitude follows a linear relationship with Young's modulus, consistent with the theory. Our results confirm that increasing the Young's modulus of PA materials is an effective strategy to increase the generated PA pressure.

To ensure the biocompatibility of devices interfacing with cells or tissues, high hydrophilicity of the surface is required. To enhance the hydrophilicity of the PDMS film, we treated it with O_2_ plasma. During this process, the methyl groups on the surface of PDMS were replaced by silanol terminal groups.^[^ ^27^ ^]^ These silanol groups form hydrogen bonds with water and thus increase the hydrophilicity of PDMS, which was confirmed by a decrease in the contact angle between PDMS and water (Figure S6, Supporting Information). Compared to unmodified PDMS, the normalized neuron viability measured by MTT on DIV 10 increased from 48.8% ± 8.6% to 95.8% ± 8% (Figure S7, Supporting Information). Thus, after surface modification, the PDMS film is biocompatible for cells or tissue cultures for biomedical applications.

### 2.3 A Multifunctional Instrument for MIPA Stimulation and Fluorescence Imaging of Neurons

**Figure 2:**
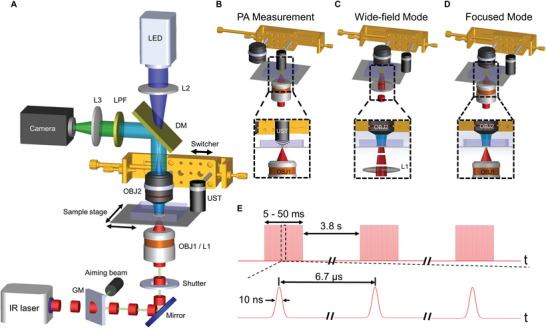
Multifunctional MIPA Optical System. A) Schematic of the system. B) Photoacoustic characterization module. Zoom‐in: focused MIR laser pulses excite the PDMS film to generate the PA wave, which is collected by a focused ultrasonic transducer (UST). The MIR beam and ultrasound transducer are confocally aligned. C) Wide‐field mode. The CaF_2_ lens weakly focuses IR beam on the sample plane for wide‐field neuron stimulation (Inset). D) High‐precision mode. A high‐NA reflective objective focuses the IR beam tightly to provide high‐precision neural stimulation (Inset). GM, germanium window. OBJ1, reflective objective. L1, CaF2 lens. UST, ultrasonic transducer. OBJ2, visible objective. DM, dichroic mirror. LPF, long pass filter. L2, L3, A‐coating lens. E) Schematic of MIR pulse train used.

A multifunctional instrument that integrates PA generation, PA measurement, and fluorescence imaging is desired for neural stimulation and activity recording. Herein, we developed a MIPA stimulation system integrating PA measurement and a wide‐field fluorescence microscope (**Figure** **2**A). Our MIPA optical system delivers the following key functions. First, in‐situ PA optimization is achieved by maximizing PA amplitude at the top surface of the PDMS via tuning confocal alignment of the optical excitation beam at the bottom of the PDMS film (Figure 2B). Second, in‐situ PA characterization is achieved by placing a hydrophone close to PDMS samples to quantify photoacoustic pressure. Third, dual optical modules offer tunable illumination areas, enabling the generation of PA in a wide‐field condition and focused condition, respectively (Figure 2C,D). The wide‐field mode of neuron stimulation is enabled by weakly focusing the IR beam on the PDMS film, while the high‐precision mode can be made by tightly focusing the IR beam via a high numerical aperture reflective objective. Fourth, the integrated system utilizes MIR‐induced PA pressure to stimulate neurons and record their fluorescence response. Specifically, we developed an optics/acoustic switcher for fast switching between the PA characterization system and wide‐field fluorescence microscope, which ensures stimulation and fluorescence recording are performed under the alignment offering the maximum PA. In addition, the duration of MIR pulse trains can be chopped from 5 to 30 ms for single‐shot and repeated simulations (Figure 2E). Using this setup, we have studied the MIPA neural stimulation with calcium imaging, as shown below.

### 2.4 Wide‐Field MIPA Neural Stimulation

A key advantage of our transparent MIPA emitter is its compatibility with wide‐field fluorescence imaging. To validate PDMS‐based MIPA stimulation of neurons in a wide‐field scheme, DIV12 primary rat cortical neurons were cultured on the top surface of the PDMS film. We weakly focused the IR beam on a 71‐µm thick PDMS film with 0.4 µJ pulse energy. The focus diameter is 200 µm, corresponding to 12.35 kPa PA pressure based on our earlier measurement (Figure 1E,H; Figure S3, Supporting Information). A 5‐ms laser pulse train with a repetition rate of 150 kHz was delivered to the bottom of the film. Before MIPA stimulation, the location of the illumination area was visualized by the aiming beam of a 532 nm CW laser (Figure 2A).

**Figure 3:**
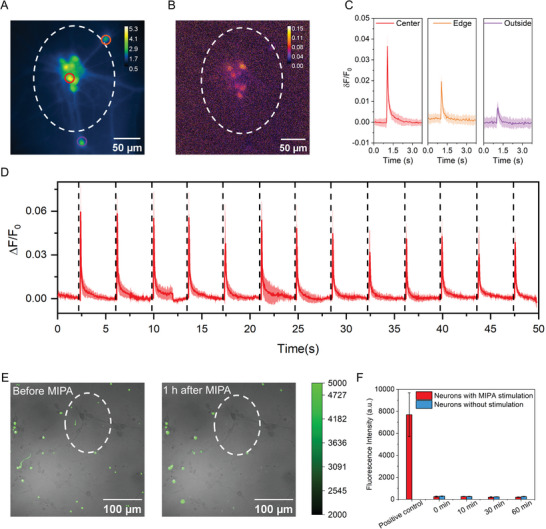
Wide‐field MIPA neural stimulation. A) A representative calcium image of Oregon green labeled cortical neurons at DIV12 cultured on the PDMS film. Dashed line: the illumination area. Solid circles: representative neurons at the center of the illumination area (red), edge of the illumination area (orange), and outside of the illumination area (purple). The inserted Color bar represents the relative fluorescence intensity. B) Map of the maximum ΔF/F_0_ of the same view field in Figure 3A. F_0_ is the average fluorescence before stimulation. Laser condition: 0.4 µJ pulse energy, 150 kHz repetition rate, 5 ms duration. C) Average calcium traces obtained from the solid circled neurons upon MIPA stimulation in Figure 3A correspondingly. Shaded areas: one standard deviation, obtained from 20‐time repeats on the same neurons. D) The average calcium trace of neurons in the illumination area in wide‐field MIPA stimulation (n = 12). Shaded areas: one standard deviation. Black dashed lines: laser onset. E) Microscope images of Sytox Green nucleus acid staining of neurons before and 1 h after wide‐field MIPA stimulation. Gray channel: bright field microscope images. Green channel: fluorescence images. Laser condition: 10 ms duration, 0.4 µJ pulse energy. White circles indicate the MIPA stimulation area. F) Comparison of fluorescence intensities of neurons stained with Sytox Green before and at different time points in both the control group and MIPA stimulation group. Before MIPA stimulation, the Sytox‐labeled nucleus of dead neurons was used as the positive control.

**Figure** **3**A,B show a map of neurons and the maximum fluorescence change ΔF/ F_0_, respectively. Here, ΔF represents the maximum fluorescence change induced by MIPA stimulation, and F_0_ is the average fluorescence intensity before MIPA stimulation. Figure 3B confirmed that the stimulation effect was confined to the MIR illumination area. Three representative calcium signal traces of neurons located at different locations are plotted in Figure 1C. The neuron in the center of the illumination area showed the largest stimulated response, with a maximum ΔF/ F_0_ of 3.6%. At the illumination edge, the stimulated response was 2%. No stimulated response was observed in the neuron outside of the illumination area. These Ca^2+^ imaging results confirm that MIPA stimulation of neurons is within the illumination area.

Repetitive stimulation was performed to confirm the safety and reliability of the MIPA stimulation by delivering 12 bursts of MIR pulses to neurons. Each burst contained a 5 ms pulse train at the same laser condition described above. As shown in Figure 3D and Figure S8 (Supporting Information), a 0.059 ± 0.018 increase in ΔF/F_0_ was observed immediately after the first MIR pulse train, suggesting successful MIPA stimulation. The acquired calcium traces of these 12 neurons showed a periodic response. At the 12th MIPA stimulation, the ΔF/F_0_ increase decreased to 0.038 ± 0.013, which can be attributed to calcium depletion,^[^ ^28^ ^]^ or spike frequency adaptation^[^ ^29^ ^]^ due to frequent stimulation. These data collectively demonstrate the reliability of MIPA stimulation.

Furthermore, we studied whether there is any potential damage induced by wide‐field MIPA stimulation using Sytox Green for nucleus staining.^[^ ^30^ ^]^ Before MIPA stimulation, the DIV12 neuron culture on PDMS was first labeled with Sytox green. Nuclei of dead neurons with compromised plasma membrane bind to Sytox Green and thus show a large fluorescence signal of an average of 7690 a.u. (n = 5) (Figure 3E,F). These initially dead neurons were used as the positive control. A laser with a 10 ms duration and 0.4 µJ pulse energy was applied for wide‐field MIPA stimulation. The Sytox fluorescence intensity of neurons in the same field of view was recorded and compared at the time points of 0, 10, 30, and 60 min after stimulation (Figure 3E,F), to evaluate potential damage induced by wide‐field MIPA stimulation. Neurons without stimulation were used as the negative control. Both neurons with and without stimulation show weak Sytox fluorescence 60 min after stimulation, only 2.3% and 3.2% of that from the positive control group, respectively (Figure 3F). This result demonstrates the negligible damage induced by MIPA stimulation.

### 2.5 MIPA Stimulation is through a Non‐Thermal Pathway

**Figure 4:**
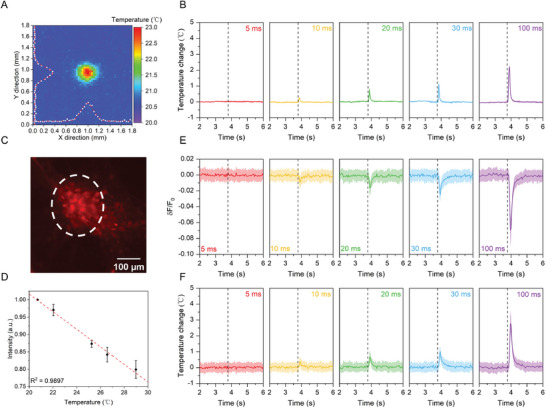
Thermal effect during MIPA stimulation. A) Temperature distribution on the top surface of PDMS after 100 ms MIR irradiation taken by a thermal camera. White dots: temperature measured along X = 1 and Y = 1, respectively. Red dashed lines: Gaussian fits of temperature measured. B) Average maximum temperature measured based on three trials with different locations of the same PDMS sample for each laser duration. Black dashed lines: MIR laser onset. C) A representative fluorescent image of mCherry labeled cortical neurons at DIV10 cultured on a 71 µm thick PDMS film. The white dashed circle: the MIR illumination area. D) Normalized fluorescence intensity of mCherry labeled cortical neurons (n = 5) at different temperatures controlled  by a dish heater. Error bars: one standard deviation of normalized fluorescence intensity of five different neurons. E) Average fluorescence intensity change of neurons (n = 52) in five different fields of view under MIR illumination areas with different MIR durations. Black dashed lines: MIR laser onset. Shaded areas: one standard deviation. F) The calculated average temperature increase of neurons in Figure 4E is based on the calibration curve in Figure 4D. Shaded areas: one standard deviation.

The MIR laser irradiation induces not only acoustic waves but also heat in the PDMS film. The photothermal effect could also modulate neural activities.^[^ ^16^, ^31^ ^]^ To quantitatively evaluate the thermal effect, we first recorded the temperature increase on the top surface of the PDMS film under the same laser condition used for successful wide‐field MIPA stimulation. We used a high‐speed thermal camera mounted above the sample stage on the MIPA optical system. Its focus was tuned to be on the top surface of the film, where neurons were cultured (Figure S9, Supporting Information). The MIR illumination area was kept at 100 µm while the thickness of the PDMS film used was 71 µm, the same as that used in wide‐field MIPA stimulation. **Figure** **4**A shows the map of the maximum temperature captured by the thermal camera after delivering a 100‐ms MIR pulse train. The center of the hot spot showed a maximum temperature of 23 °C, a 3 °C increase from the background temperature. The shape of the hot spot showed a Gaussian distribution (Figure 4A), which is consistent with the Gaussian shape of the MIR beam. Temperature changes at the center of the hot spot on the top surface of the film were also measured with different MIR pulse laser durations. As shown in Figure 4B, the peak temperature decreased dramatically when the duration decreased. With a 10 ms MIR duration, the peak temperature increase (ΔT) was only 0.25 ± 0.1 °C, which is much lower than the typical temperature increases required for photothermal neural modulation.^[^ ^16^, ^32^ ^]^ Importantly, no temperature increases on the top surface of PDMS films were detected after 5 ms MIR duration, the condition for successful MIPA stimulation.

To directly measure the temperature inside the neuron, we transfected neurons with mCherry (Figure 4C), a temperature‐sensitive fluorescent protein,^[^ ^33^ ^]^ to monitor the temperature change of neurons under MIPA stimulation. First, we measured the fluorescence intensity change of mCherry in neurons at different temperatures controlled by a dish heater. The temperature‐dependent fluorescence curve plotted in Figure 4D shows that a 1 °C temperature increase leads to ≈2.5% fluorescence intensity decrease. Next, we used the fluorescence intensity change in mCherry to quantify the temperature change of neurons under MIPA stimulation. As expected, the fluorescence intensity of mCherry in the neurons showed a significant ΔF/F_0_ of 0.069 ± 0.019 after 100 ms MIR irradiation. The ΔF/F_0_ showed 0.025 ± 0.008, 0.016 ± 0.011, and 0.009 ± 0.008, respectively, for the 30, 20, and 10 ms MIR irradiation. With 5 ms irradiation, no fluorescence decrease was observed. Based on the calibration curve, these fluorescence changes correspond to temperature increases of 2.76 ± 0.76 °C, 1.00 ± 0.32 °C, 0.64 ± 0.44 °C, and 0.36 ± 0.32 °C for 100 ms, 30 ms, 20 ms, and 10 ms, respectively (Figure 4F). Specifically, with 5 ms MIR irradiation, the condition used for successful wide‐field MIPA stimulation, temperature increases in neurons are negligible, consistent with the temperature measurement by the thermal camera. Together, our data indicate a non‐thermal mechanism for MIPA stimulation.

### 2.6 High‐Precision MIPA Stimulation of Single Neurons

**Figure 5:**
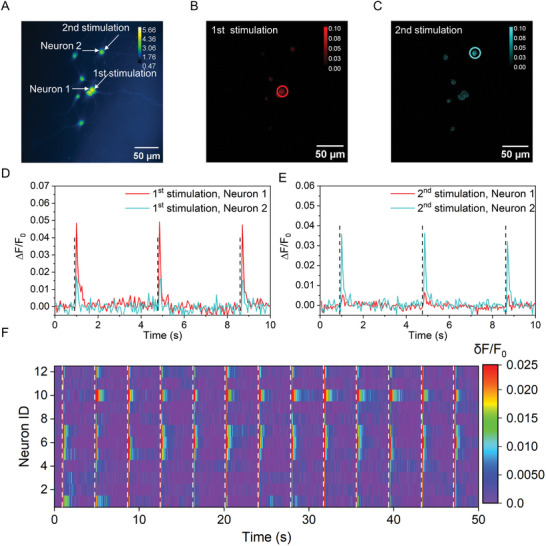
High‐precision MIPA neural stimulation. A) Representative calcium image of Oregon Green labeled cortical neurons at DIV12 cultured on the PDMS film before sequential MIPA stimulation. White arrows indicate the targeted neurons. The color scale represents relative fluorescence intensity. B,C) Maps of the maximum ΔF/F_0_ induced by two sequential MIPA stimulations. Red: the first stimulation. Cyan: the second stimulation. Laser condition: 0.1 µJ pulse energy, 150 kHz repetition rate, 15 ms duration. Solid circles: 1st stimulated area (red) and 2nd stimulated area (cyan). Color scales represent ΔF/F_0_. D–E) Corresponding calcium traces of Neuron1 and Neuron2 during two sequential MIPA stimulation in Figure 5B,C. Black dashed lines: the laser onset. D) the first MIPA stimulation. E) the second MIPA stimulation. F) The heatmap of fluorescence change in neurons cultured on the PDMS film under the illumination area. White dashed: the onset of the laser.

A key advantage of PA stimulation over ultrasound is its high spatial resolution for single neuron or subcellular stimulation versus hundreds of micron for traditional ultrasound neural stimulation,^[^ ^10^, ^20^, ^34^ ^]^ which is important for studying information flow in neuronal networks. Since a tightly focused MIR laser can generate an illumination area of 10 µm, we explored the possibility of high‐precision MIPA stimulation using the focused mode. The MIR beam was tightly focused by a 0.5‐NA reflective objective. The diameter of the IR focus spot was measured as ≈10 µm. To avoid enlarging the PA field during acoustic propagation, we cultured neurons on a thin PDMS film with a thickness of 25 µm (**Figure** **5**A). A manual 2D stage was used to precisely match the IR focus and the targeted neuron in the lateral plane.

With 0.1 µJ pulse energy and 15 ms burst duration, a PA pressure of 696 kPa was delivered to the top surface of PDMS according to the plotted pressure‐distance relation. A clear Ca^2+^ signal increase was observed immediately after each laser pulse train delivery, indicating successful neural stimulation (Figure 5B,C). In the two sequential MIPA stimulations, only the targeted neurons showed a significant response, with ΔF/F_0_ ≈4%, to the MIR laser, while neurons located ≈50 µm away remained unstimulated (Figure 5B–E), confirming the high spatial resolution in this focused mode. Furthermore, 12 targeted neurons showed similar transient and repetitive responses to MIPA stimulation (Figure 5F). Therefore, our results demonstrate the ability of MIPA stimulation to achieve single‐neuron stimulation without affecting the viability of neurons.

### 2.7 Wide‐Field MIPA Stimulation Distinguishes Neurons Overexpressed with Different Ion Channels

**Figure 6:**
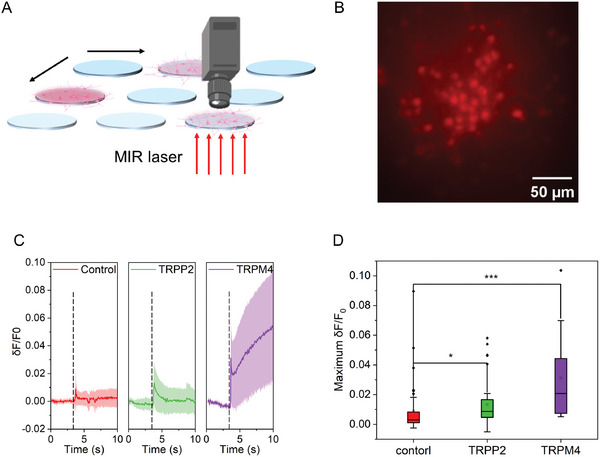
Neurons overexpressed with different ion channels show distinct responses under wide‐field MIPA stimulation. A) Schematic of wide‐field MIPA stimulation based high throughput cell screening. MIR laser was weakly focused on the bottom surface of PDMS and a 2D stage was used to control the targeted wells. B) A representative fluorescence image of mCherry labelled, TRPM4 overexpressed cortical neurons in DIV 12. C) Average calcium traces of wild‐type neurons (red, n = 40), TRPP2 overexpressed neurons (green, n = 38), and TRPM4 overexpressed neurons (purple, n = 14) cultured on 71 µm thick PDMS films. Laser condition: 0.2 µJ pulse energy, 150 kHz repetition rate. The shaded areas: one standard deviation. Black dashed: laser onset. D) Comparison of the maximum fluorescence intensity changes obtained in Figure 6C. (n > 14, *p < 0.05, ***p < 0.001, n.s. p = 0.147, Kruskal‐Wallis ANOVA test.).

Ion channels expressed on neurons are found to be responsible for acoustic neural stimulation.^[^ ^5a^ ^]^ Specifically, Shapiro et.al revealed that ultrasound neural stimulation is mediated through calcium‐selective mechanosensitive ion channels.^[^ ^33b^ ^]^ As a transparent PA emitter with the capability to induce safe and reliable neural stimulation, wide‐field MIPA stimulation can potentially be used as a screening tool (**Figure** **6**A). As a proof‐of‐concept demonstration, we studied the roles of two different ion channels, TRPP2 and TRPM4, during wide‐field MIPA stimulation. We overexpressed these two ion channels in Oregon Green labeled neurons under a hSyn promoter. Here, mCherry was co‐expressed to confirm the successful labeling (Figure 6B). Then, 10 ms wide‐field MIPA stimulation was performed on three different groups of neurons, including wild type, TRPP2 overexpressed, and TRPM4 overexpressed neurons. The same illumination area was used in wide‐field MIPA stimulation to record the response in different groups. In each group, ≈10 neurons (neuron density: 300 cells mm^2^) were stimulated and their Oregon Green fluorescence intensities were recorded simultaneously.

As shown in Figure 6C,D, the TRPP2 overexpressed and TRPM4 overexpressed neurons showed the average ΔF/F_0_ of 0.013 ± 0.015 and 0.032 ± 0.029, respectively, upon 10 ms MIPA stimulation, while wild‐type neurons showed only 0.009 ± 0.016 ΔF/F_0_. This result confirms that these two ion channels play an important role in photoacoustic stimulation. The involvement of these specific mechanosensitive channels in photoacoustic stimulation could be similar to that reported in ultrasound stimulation.^[^ ^33b^ ^]^ Together, these studies indicate that wide‐field MIPA stimulation and fluorescence imaging on a single setup can be used as a high‐throughput screening platform for ion channels and modulators involved in ultrasound neuromodulation.

## 3 Conclusion

We have reported a new photoacoustic emitter based on mid‐IR excitation of C‐H bonds in PDMS. Although vibrational excitation has a smaller absorption cross‐section compared to electronic excitation, the higher concentration of C─H bonds in PDMS makes it a stronger absorber than the carbon nanomaterials embedded in the PDMS matrix. The uniqueness of generating photoacoustic through vibrational excitation allows the photoacoustic emitter to be optically transparent in the visible and near‐infrared window. We showed that the PDMS film emits a broadband acoustic wave with an initial 58 kPa peak‐to‐peak pressure and 10 µm spatial resolution by a 0.2 µJ nanosecond MIR laser pulse at the wavelength of 3.38 µm. The photoacoustic conversion was found to be 37.5 times more efficient than the carbon nanotube‐based PA film upon visible excitation due to bypassing the thermal conduction in PA generation. We further optimized PA conversion efficiency by increasing the Young's modulus of the PA materials. A 26% enhancement of PA conversion was achieved with a 6:1 base‐to‐agent ratio PDMS film compared to the 10:1 base‐to‐agent ratio PDMS film used previously.

Toward applying PDMS‐based MIPA in biomedical applications, specifically in neural stimulation, we developed several key enablers. Through surface modification with O_2_ plasma, the hydrophilicity and biocompatibility of the PDMS film were improved substantially. O_2_ plasma‐treated PDMS was shown to be a viable substrate to culture cortical neurons. We also built a multifunctional MIPA optical system performing PA characterization, fluorescence imaging, and neural stimulation with a tunable stimulation area.

Both wide‐field and high‐precision neural stimulation were achieved in the range between 12.35 to 696 kPa MIPA pressure with a tunable stimulation area from 50 to 200 µm diameter. PDMS‐based MIPA was shown to be repeatable and reliable in neuron culture, evident from >10 times repeated stimulation on the same field of view. No damage was observed 60 min after 10 ms wide‐field MIPA stimulation, confirmed by Sytox Green nucleus staining.

Furthermore, PDMS has a thermal conductivity of 0.16 Wm^−1^ K^−1^, much lower than both water and the CaF_2_ substrate, 0.6 and 8.6 Wm^−1^ K^−1^ respectively. In the current configuration of MIPA stimulation, PDMS was placed on CaF_2_ substrates with an MIR pulsed laser delivered to the PDMS bottom surface while neurons cultured on its top surface. Therefore, the generated heat at the bottom surface of PDMS is mainly dissipated into CaF_2_ due to the larger thermal conductivity in CaF_2_. Evidently from the measurements done with the thermal camera and mCherry reporting, a negligible temperature increase was observed under 5 ms wide‐field MIPA stimulation conditions. The thermal damage to neurons cultured on the top surface was prevented. In other in vitro configurations without the presence of the CaF_2_ substrate, a cooling strategy such as a cooling fan is needed.

Being transparent in the visible and NIR range, PDMS‐based MIPA neural stimulation provides a new platform to study cell responses to mechanical stimuli using bioimaging as a readout. Importantly, wide‐field stimulation and imaging enable high‐throughput cell screening. As a proof of concept, we studied the roles of two different ion channels (TRPP2, TRPM4) in MIPA stimulation using the wide‐field capability. Owing to the high repeatability and reliability of MIPA, the response of a large number of neurons (n = 92 in total) was recorded by simply positioning the MIR beam in different wells in a 6‐well plate. The current area of wide‐field stimulation is limited by the power of the laser available in the lab. With a commercially available high‐energy MIR OPO laser, the pulse energy can reach up to several mJ.^[^ ^35^ ^]^ A 2500 times bigger illumination area corresponding to 5 mm diameters can be generated with a similar energy density and result in similar PA pressure. Considering 300 cells mm^2^ cell density, over 20 000 neurons in a 5 mm area can be stimulated in single wide‐field MIPA stimulation to achieve high‐throughput screening. This pilot study also offers some new insights into the mechanism of photoacoustic neural stimulation. We demonstrated that mechanosensitive ion channels such as TRPP2 and TRPM4,^[^ ^33^, ^36^ ^]^ are responsible for MIPA neural stimulation by showing the enhancement of MIPA stimulation through overexpressing these ion channels.

C─H bonds are widely present in organic compounds. Our PDMS‐based MIPA provides a universal bond selective method to efficiently generate PA. Our work confirmed the feasibility of achieving the PA stimulation of neurons through vibrational excitation of the C─H bonds in PDMS as a PA emitter. In principle, organic molecules and films can be used as bond‐selective PA emitters by selectively exciting the C‐H bonds with a pulsed MIR laser.

Given the fact that water has a large absorption at 3.38 µm, to avoid the MIR heating of water, the MIR beam used for photoacoustic stimulation can't be delivered to the same side of the PDMS where neurons are cultured. This requirement limits the application of current MIPA stimulation in vivo. However, PDMS can be used as an optically transparent transcranial window^[^ ^37^ ^]^ to receive MIR irradiation on one side of PDMS window and generate PA pressure on the other side of PDMS without heating. In that way, MIPA stimulation of cortex in animal models can be achieved.

## 4 Experimental Section

### Fabrication of PDMS Films

PDMS (Sylgard 184, Dow Corning Corporation) was used without further purification. To fabricate the films, 1 g PDMS was mixed with the desired base‐to‐cure agent ratios in the range of 1: 1 to 10: 1. The mixture was then placed in a vacuum chamber to degas for 30 min. After degassing, 1 mL mixture was poured onto a 30 mm diameter CaF_2_ substrate for further spin‐coating (Karl Suss, Delta 80T2/200) for 5 min. Rotating speeds in the range of 200 to 2200 rpm gave the thickness of the PDMS film in the range of 90 to 5 µm. Finally, the PDMS‐coated CaF_2_ substrates were placed on a 120 °C hotplate for 5 min to cure the PDMS.

### Characterization of PDMS Films

The FTIR spectrum was collected by a Bruker Optics Vertex 70v FTIR instrument. The thicknesses of PDMS films were determined by the Alpha Step 500 surface profiler (KLA Tencor). The UV–vis absorption spectrum was measured by a UV–vis spectrometer (UV‐1900i, Columbia, MD, USA). The Young's modulus of the PDMS film was measured on an Instron 5944 Micro‐tester.

### Photoacoustic Measurement

To generate the PA wave, a 10‐ns mid‐infrared laser for PDMS film (Firefly IR SW, M squared inc. UK. Pulse repetition rate 150 kHz, maximum pulse energy 0.4 µJ) or a 2.6‐ns green laser for CNT‐PDMS film (Bright Solutions, Pavia, Italy. Pulse repetition rate 150 kHz, wavelength 532 nm, pulse energy ranges from 0.02 to 0.2 µJ) was focused on the samples through a reflective objective (LMM40XF‐P01, Thorlabs) or a 10X objective with the illumination area ≈10 µm. The energy delivered to the sample was measured by an optical power meter (PM100D, Thorlabs). An ultrasound transducer (25 MHz, V324‐SM, Olympus) was used to detect the generated acoustic waves. After being amplified by a 40 dB amplifier (SA‐251F6, NF Corporation Inc.), the generated PA signal was sent to a data acquisition board (EON Express, Vitrek) to be analyzed and displayed. During the experiment, both films and the transducer were immersed in deionized water. A 40 µm diameter hydrophone (Precision Acoustics, Dorchester, UK) was used to measure the pressure of the PA wave generated by PDMS films 600 µm away from the PDMS.

### Pretreatment of the PDMS Film for Neuron Culture

Before culturing neurons, the wettability of PDMS was modified using a previously reported O_2_ plasma method.^[^ ^38^ ^]^ 2 days before seeding neurons, the fabricated PDMS films were exposed to O_2_ plasma for 1 min with a power of 100 W (M4L RF plasma system, PVA TePla American) followed by 60 min UV light sterilizing. After that, the treated PDMS films were immersed in 50 µg mL^−1^ poly‐D‐lysine (PDL) solution overnight to facilitate the adhesion of neurons. One day before neuron seeding, the PDMS films were immersed in the seeding medium containing 90% Dulbecco's modified Eagle medium (ThermoFisher Scientific) and 10% fetal bovine serum (ThermoFisher Scientific) and placed in a CO_2_ incubator.

### Embryonic Neuron Culture

All experimental procedures complied with all relevant guidelines and ethical regulations for animal testing and research established and approved by Institutional Animal Care and Use Committee (IACUC) of Boston University (PROTO201800534). Primary cortical neurons were isolated from Sprague‐Dawley rats (Charles River Laboratory) on embryonic day 18 (E18) of either sex and digested in TrypLE Express (ThermoFisher Scientific). Neurons were first plated on the treated PDMS films in the dishes with the seeding medium containing 90% Dulbecco's modified Eagle medium (DMEM, ThermoFisher Scientific) and 10% fetal bovine serum (FBS, ThermoFisher Scientific). After 24 h culturing, the growth medium composed of Neurobasal medium (ThermoFisher Scientific) with 2% B27 (ThermoFisher Scientific), 1% N2(ThermoFisher Scientific), and 2 mM GlutaMAX (ThermoFisher Scientific) was used to replace the seeding medium. Half of the medium was replaced by fresh growth medium every 3–4 days. After 10–14 days, Oregon green 488 BAPTA‐1 (ThermoFisher Scientific) dissolved in 20% wt F127‐DMSO solution was applied to neurons with the final concentration of 2 µM for 30 min and followed by 30 min incubation in culture medium before MIPA stimulation. The viability of the cultured neurons culturing on PDMS was determined using the MTT assay (Thermo Fisher Scientific).

### Wide‐Field Fluorescence Imaging

A wide‐field fluorescence microscope was used to record fluorescence from Oregon Green‐labeled neuron's response. A 470‐nm LED source (M470L5, Thorlabs) was used as the excitation source. An achromatic doublets lens (AC254‐075‐A‐ML) was used to focus the excitation laser on the back focal plane of the imaging objective (LUCPlanFL N 20X, Olympus). A dichroic mirror (MD498, Thorlabs) was used to combine the excitation path and fluorescence collection path. The fluorescence signal was further filtered by a longpass filter (FELH0500, Thorlabs) to remove autofluorescence as focused by a tube lens (ITL200, Thorlabs) to a real‐time imaging camera (Grasshopper 3, FLIR).

### In Vitro Neuron Stimulation

Cortical neurons were cultured on PDMS films as described in *Embryonic Neuron Culture*. The 10 ns mid‐infrared laser with a 150 kHz repetition rate (Firefly IR SW, M squared inc. UK) with desired pulse energy was tightly focused on the bottom of the PDMS film through a reflective objective for the focus mode, or weakly focused with a CaF_2_ lens to achieve 100 µm illumination area for the wide‐field mod. A 532 nm green laser beam was aligned with the mid‐infrared laser beam as the aiming beam to indicate the position and the illumination area of the mid‐infrared laser spot. The culture dish was placed on a microscope stage which can be controlled by a 3D micromanipulator (Thorlabs, Inc., NJ, USA) to target the laser beam to a specific area of neurons.

Calcium imaging was performed on the lab‐build wide‐field fluorescence microscope described in the *Calcium imaging microscope*. The acquired fluorescence data and curve fitting were further analyzed by ImageJ (Fiji) and Origin 2018.

### Sytox Green Nucleus Acid Staining

A 5 mM SYTOX Green nucleic acid stain solution in DMSO (S7020, ThermoFisher Scientific) was added in the neuron culture dish with a final concentration of 1 µM 10 min before the MIPA stimulation.^[^ ^30^ ^]^ Before the PA stimulations, the nucleus of some neurons constantly emitted green fluorescence signals, and these neurons were identified as the dead neuron group. For the MIPA‐stimulated neurons, in situ Sytox fluorescence images were captured before, 10 min after, 30 min after, and 60 min after 10 ms wide‐field MIPA stimulations. The fluorescence intensities of stimulated neurons were compared to confirm their viability.

### Temperature Measurements during MIPA Stimulation

Two temperature measurements were performed. In the measurement done with the thermal camera, the MIR laser with a 0.4 µJ pulse was weakly focused on the bottom surface of the PDMS film with an illumination area of 100 µm. A thermal camera (TELEDYNE FLIR A300) was focused on the top surface of PDMS films to record the temperature increase with MIR irradiation durations of 5 – 100 ms.

For temperature‐sensitive mCherry imaging, neurons in DIV12 were first transfected with pAAV‐hSyn‐mCherry (Addgene) on day 5 with a final concentration of 1 µg mL^−1^ to express mCherry. The same wide‐field fluorescence imaging system as previously described was used to detect the mCherry fluorescence signal. A 565 nm LED source (M565L3, Thorlabs) was used as the excitation source.

To obtain the calibration curve, the neuron culture was heated to different temperatures by a dish heater and the temperature was monitored by an electrical thermal probe in the medium. The mCherry fluorescence signal was recorded when the temperature was stable with a fluctuation of less than ±0.2 °C in 2 min. A sequence of images over 100 frames was taken at each temperature to correlate the fluorescence intensity to the temperature measured by the thermal probe. Fluorescence intensity was normalized by the average intensity obtained at room temperature.

To measure the mCherry fluorescence change upon MIPA stimulation, no dish heater was used. The MIR laser (Firefly IR SW, M squared inc. UK.) at 3.38 µm wavelength,0.4 µJ pulse energy was weakly focused at the bottom surface of PDMS through a CaF_2_ lens with durations of 5–100 ms. The MIR laser was delivered at t = 3.8 s and a total of 10 s video was acquired to record the fluorescence change of mCherry in neurons cultured on the top surface of PDMS.

### Gene Overexpression of Ion Channels in Cultured Neurons

The viral particles expressing TRPP2 and TRPM4 are obtained from Mikhail G. Shapiro's group at California Institute of Technology. As described in their previous work,^[^ ^12a^ ^]^ the mouse TRPP2 (GenBank: BC053058) and TRPM4 (GenBank: BC096475) genes were synthesized commercially (Integrated DNA Technologies) and cloned upstream of an internal ribosome entry site (IRES2) and mScarlet (TRPP2) or mRuby3 (TRPM4) gene. The construct was inserted into the lenti‐backbone. The viral particles were added to neurons at 3 days in vitro (1E9 vp/sample) and maintained for 10 days. hSyn‐driven mCherry was inserted into the lenti‐backbone by Gibson assembly to confirm the gene expression.

## Conflict of Interest

C.Y. and J.X.C. serve as Scientific Advisor for Axorus. CY received a research grant from Axorus, which did not support this work. C.Y. and J.X.C. have a patent on Methods and Devices for Optoacoustic Stimulation (US Patent No. 11684404 B2) issued.

## Author Contributions

Z.D. and M.L. contributed equally to this work. C.Y. and J.X.C. initiated the idea and guided the project. Z.D. performed the fabrication and characterization of materials. Z.D. prepared the neuron cultures. M.L. designed and built the photoacoustic and fluorescence microscope instrument. Z.D. and M.L. performed the stimulation and recording experiments of neurons. G.C. helped with the neuron stimulation and recording experiments. M.X. and Z.D. measured Young's modulus of the materials. D.J. and Z.D. performed the FTIR spectrum measurement of materials.

## Supporting information

Supporting Information

## Data Availability

The data that support the findings of this study are available from the corresponding author upon reasonable request.
