# Supplementary material for: Mid‐Infrared Photoacoustic Stimulation of Neurons through Vibrational Excitation in Polydimethylsiloxane
Source: Adv Sci (Weinh). 2024 Jul 12;11(35):2405677. doi: 10.1002/advs.202405677 (PMC11425203; doi:10.1002/advs.202405677)
Supplement: Supplementary file 1 — Supporting Information [file ADVS-11-2405677-s001.docx]

Supporting Information

**Mid-infrared Photoacoustic Stimulation of Neurons Through Vibrational Excitation in Polydimethylsiloxane**

*Zhiyi Du^1†^, Mingsheng Li^2†^, Guo Chen^2^, Maijie Xiang^3^, Danchen Jia^2^, Ji-Xin Cheng^2,4*^, Chen Yang^1,2*^*

*Fabrication of CNT-PDMS films:* The CNT-PDMS film was fabricated according to the previously reported method ^1^. CNT powders (<8 nm OD, 2–5 nm ID, length 0.5–2 μm, VWR, Inc., NY, USA) were mixed with PDMS solution (base to agent ratio 6:1) using 300 rpm magnetically stirring with the mass concentration of 10% in isopropanol. After 30 min of vacuum degassing, 1 mL uniform mixture was poured onto a 20 mm length rectangular cover glass for further spin-coating. After 1800 rpm spin-coating for 20 s, the CNT-PDMS coated cover glass was transferred to a 120 °C hotplate for 5 min to cure the PDMS.

*Photoacoustic conversion efficiency comparation:* The photoacoustic conversion efficiency is calculated according to the following equation ^2^:

$$K=\frac{\frac{1}{T}\int│P\left( T \right)│dt}{\frac{1}{t}\int I\left( t \right)dt}$$

where P(T), I(t), T and t are the pressure, the optical intensity, and the temporal period for the ultrasound and the laser pulse respectively. Since the pressure is proportional to the voltage detected by transducer, the voltage is used in the equation to quantitively compare the PA conversion efficiency. The comparation of PA conversion efficiency of different samples are described as the following equations:

$$\frac{K_{1}}{K_{2}}=\frac{(\frac{1}{T}\int{│V}_{1}\left( T \right)│dt )(\frac{1}{t}\int I_{2}\left( t \right)dt)}{(\frac{1}{t}\int I_{1}\left( t \right)dt)(\frac{1}{T}\int{│V}_{2}\left( T \right)│dt)}$$

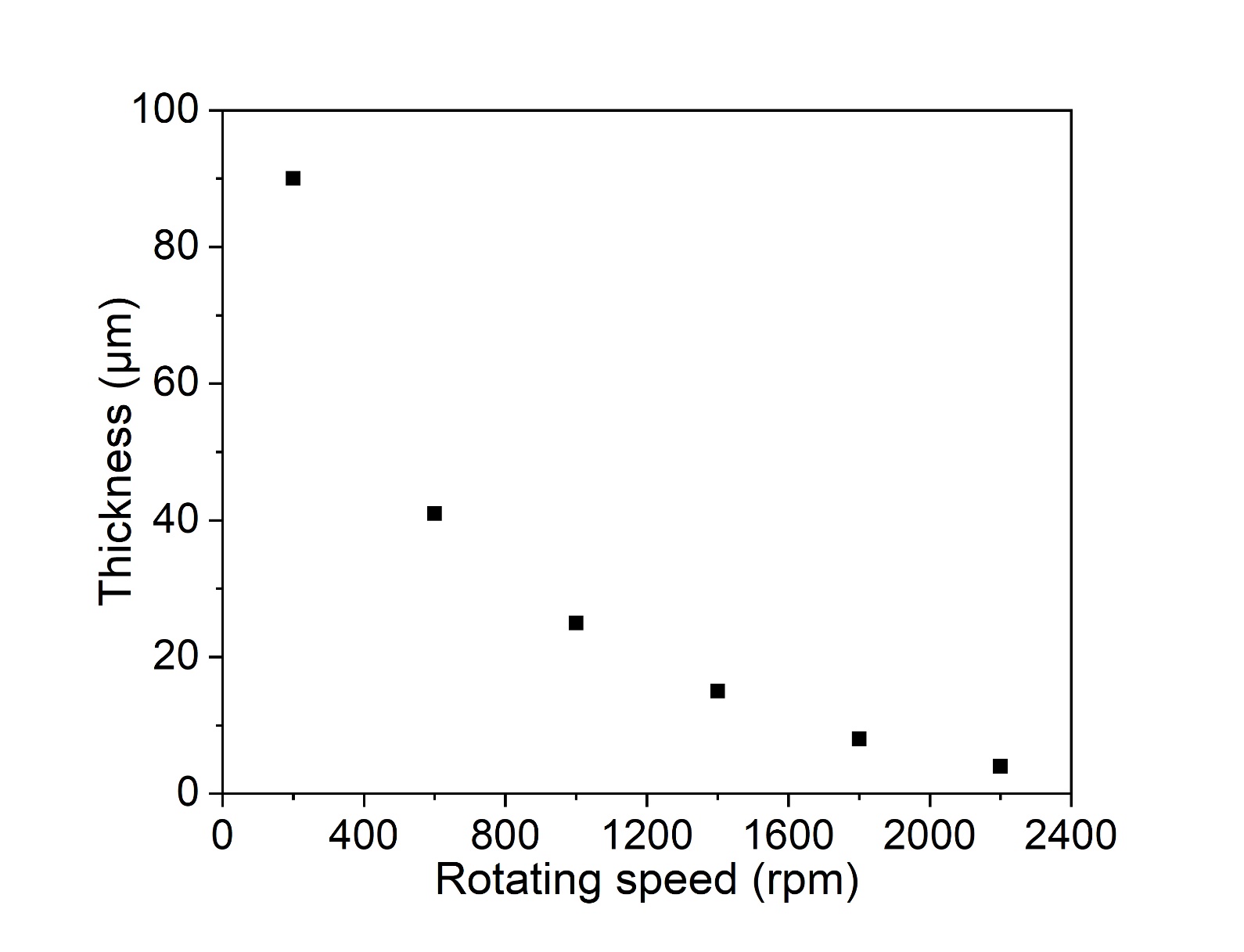


**Figure. S1.** Thicknesses of PDMS films fabricated by different spin-coating speeds.


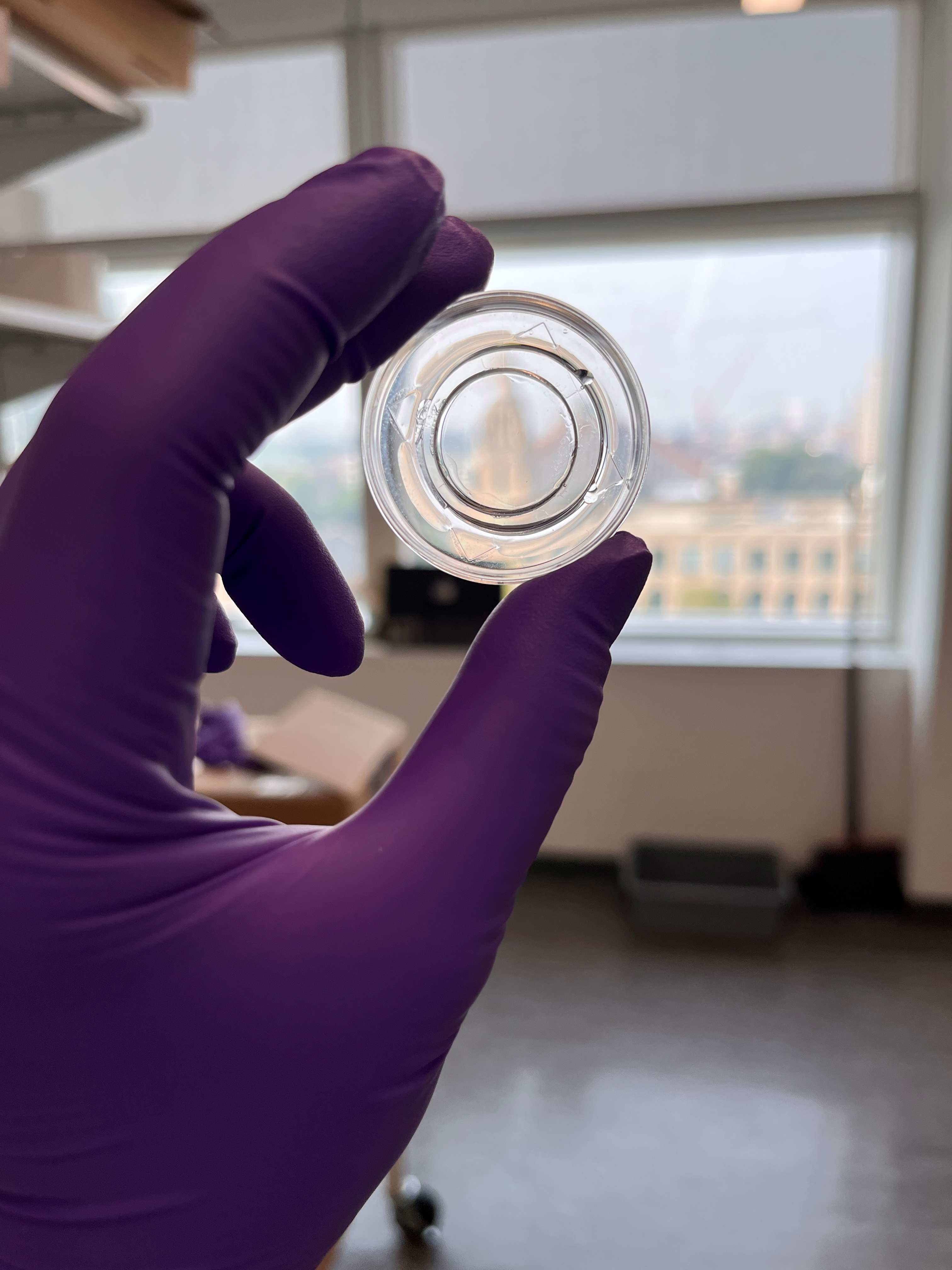


Figure. S2. The photo of a 15 μm PDMS film coated on CaF_2_ substrate.


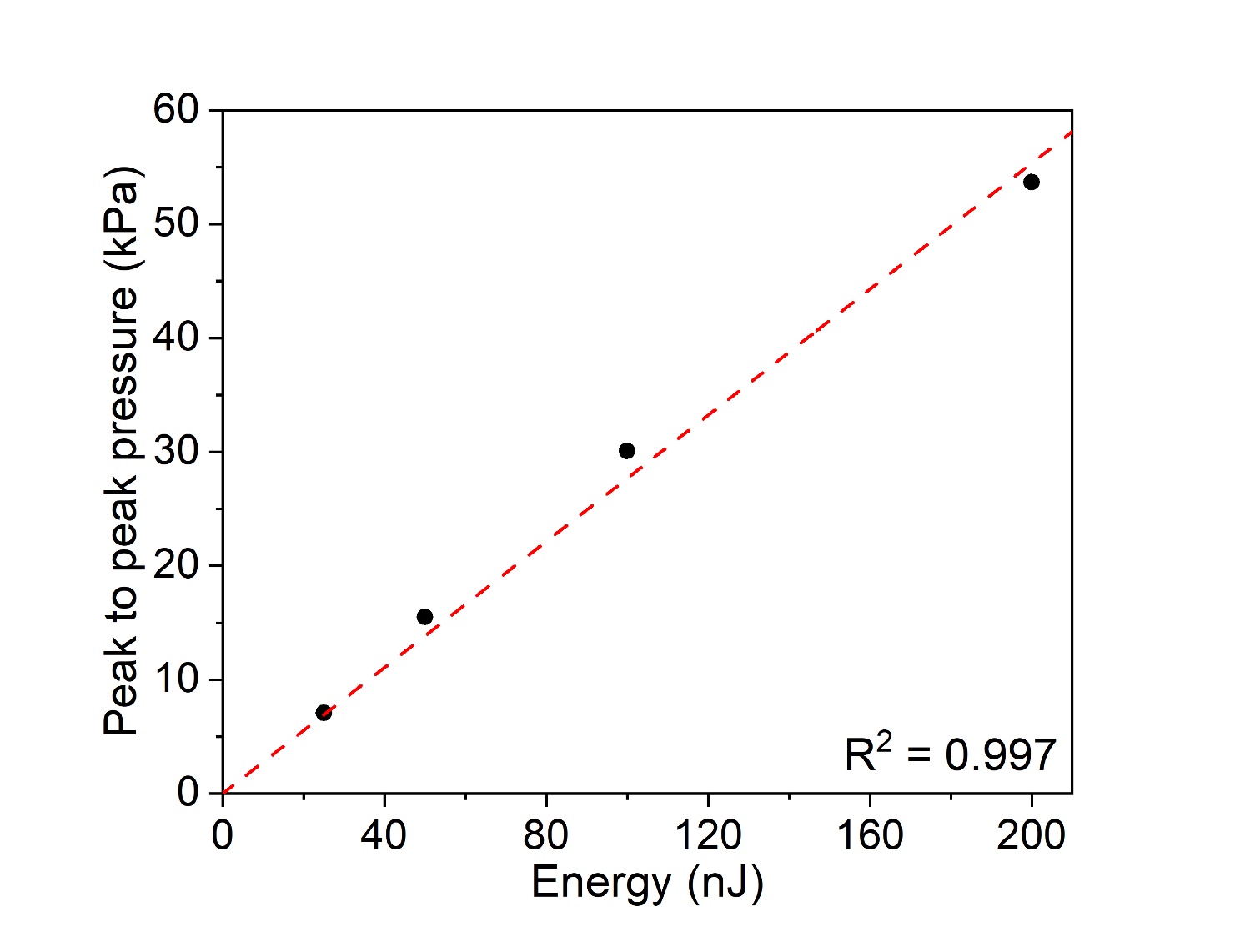


Figure. S3 Peak to Peak PA pressures measured at different laser pulse energies. Black dots: experiment data. Red dashed line: linear fitted curve.


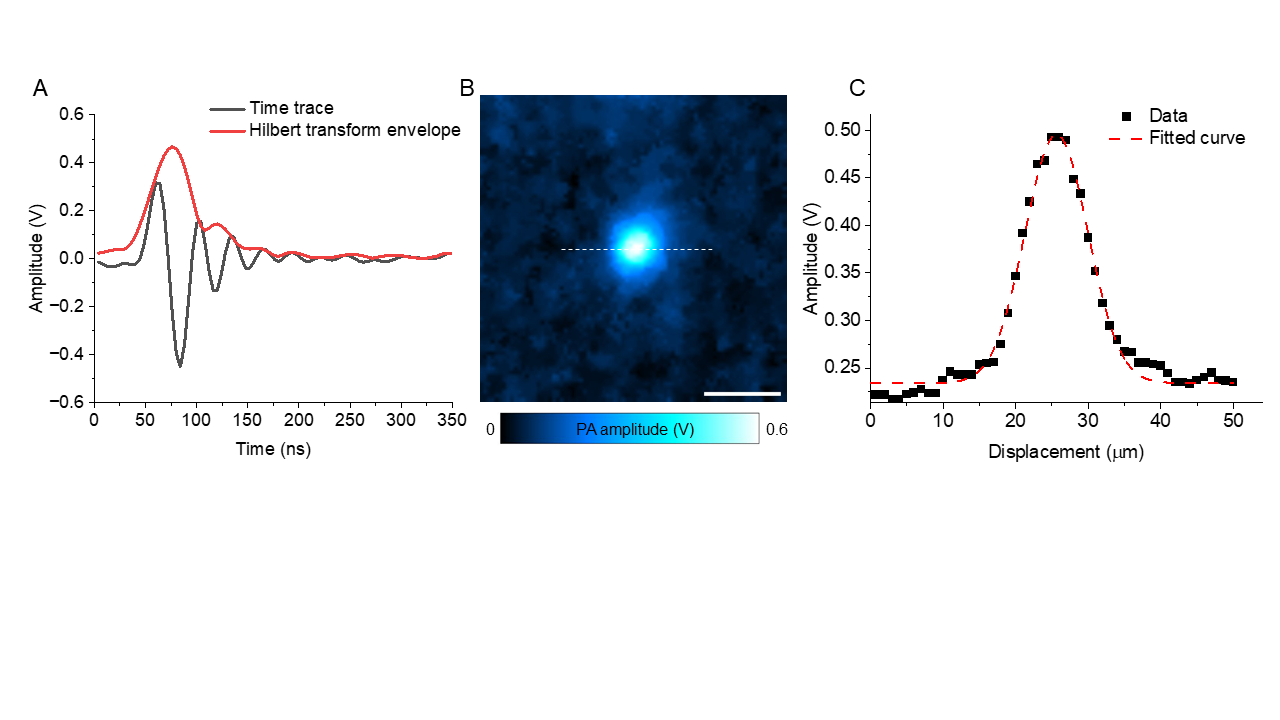


Figure. S4: Visualization of ultrasound field generated in the focused mode via MIPA microscopic imaging of C-H bonds in samples. (A) The PA waveform generated. A Hilbert transform envelope is included as it will be used to generate the contrast in the PA image in Figure S4B. Laser conditions: 10 ns pulse width, 3.41 μm wavelength. (B) The PA image obtained in the focused mode. The peak-to-peak amplitude of the Hibert transform envelope was used as PA intensity at each pixel. Scale bar: 20 µm. In the imaging system, the tight IR focusing beam was kept still and the ultrasonic transducer was placed within water medium above the sample plane. Then, a 2D translation stage (Mad City Labs, Nano-Bio 2200) was used to scan the poly(methyl methacrylate) (PMMA) beads and the PA time-resolved signal was recorded at each pixel. (C) Intensity trace along the dash line in Figure S4B. Red dashed curve: Gaussian fitted curve. The full-width-at-half-maximum of the intensity trace is 10.2 μm, indicating the diameter of the photoacoustic field. A representative PA time trace was shown in Figure S4A (black curve). The sample measured was a 1 μm PMMA bead.


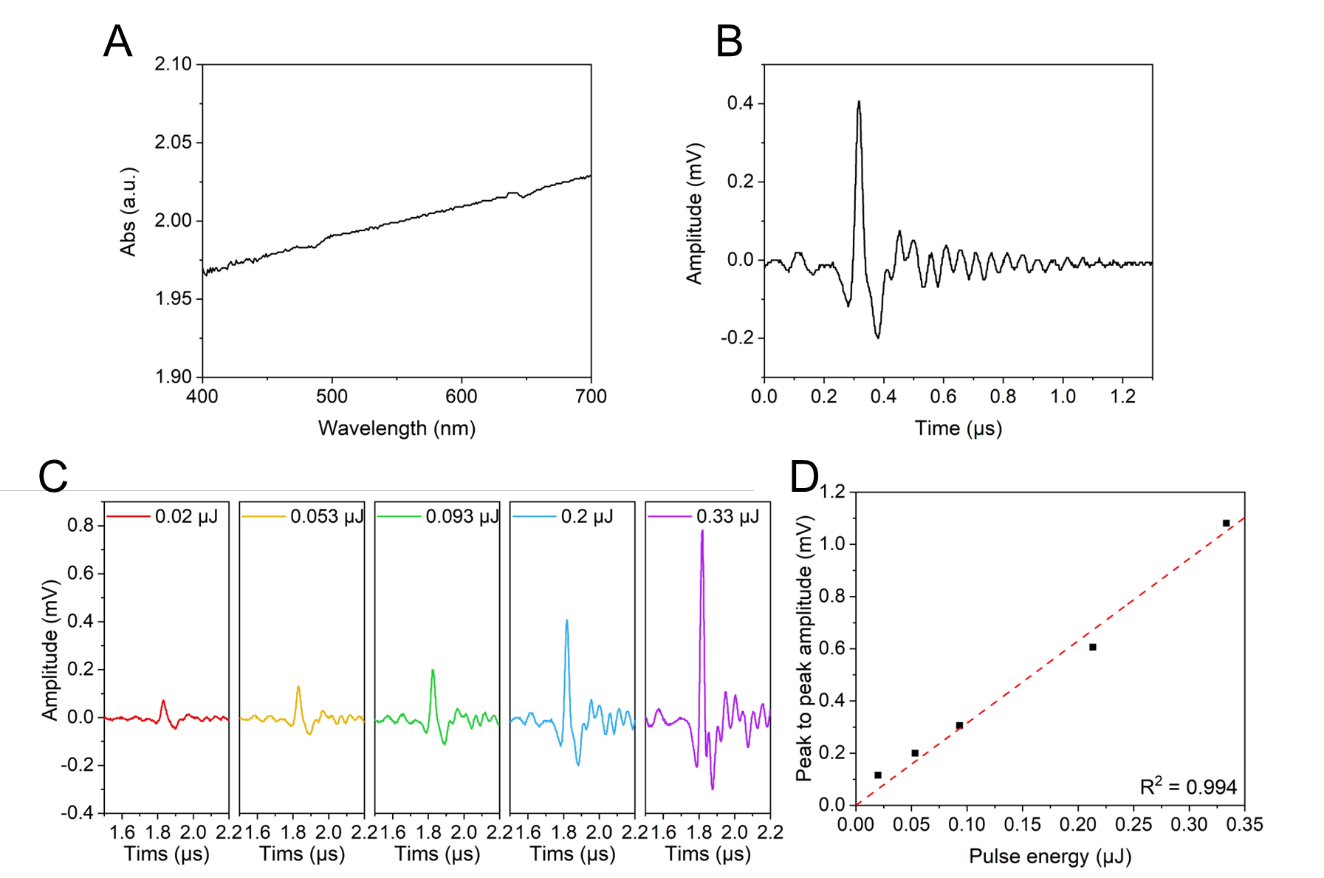


Figure. S5: Characterizations and PA measurements of the CNT-PDMS film. (A) UV-Vis absorption spectrum of the CNT-PDMS film. (B) PA waveform generated by the CNT-PDMS film with 0.2 μJ pulse energy measured by the 25 MHz transducer. (C) PA waveform generated by the CNT-PDMS film with different pulse energies. (D) Peak to Peak PA amplitude measured at different laser pulse energies. Black dots: experiment data. Red dashed line: linear fitted curve. Laser condition: 532 nm wavelength, 2.6 ns pulse width,


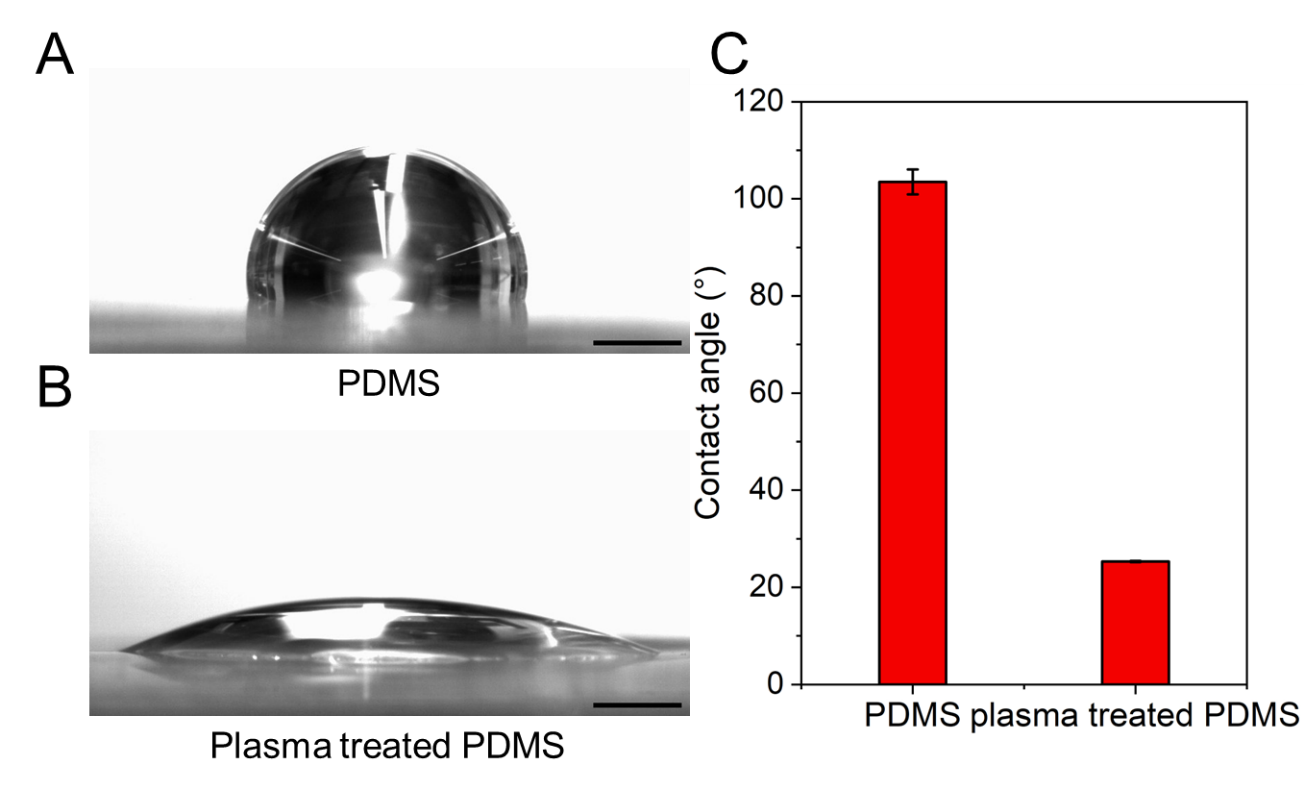


Figure. S6. (A), (B). Photos of water contact angles of the untreated PDMS film (A) and the plasma treated PDMS film (B). (C). The comparison of water contact angles of the untreated PDMS film and the plasma treated PDMS film.


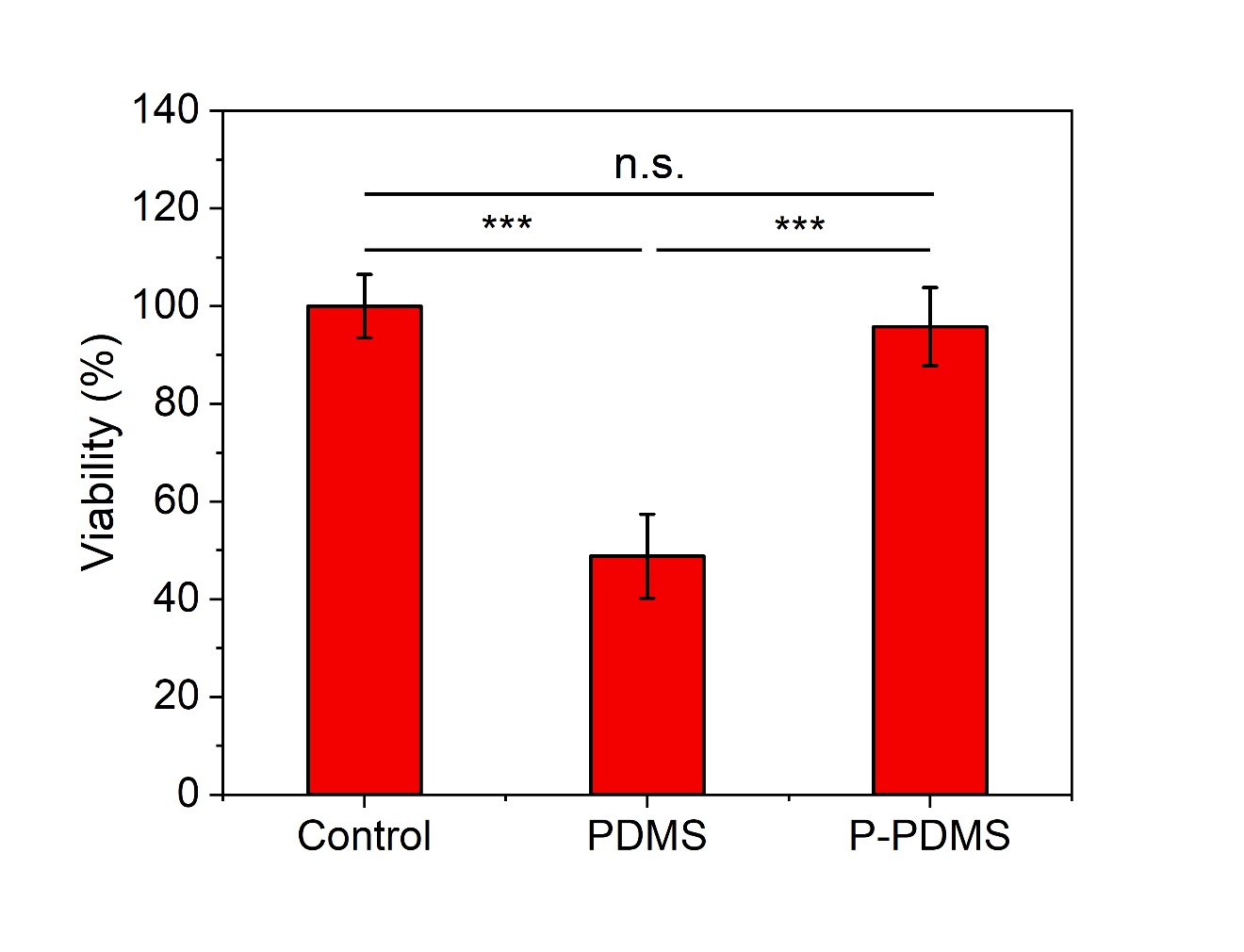


Figure. S7. Cell viabilities of primary cortical neurons on DIV 10 cultured on different samples. The viability is measured by MTT assay (n = 4). P-PDMS: plasma treated PDMS.


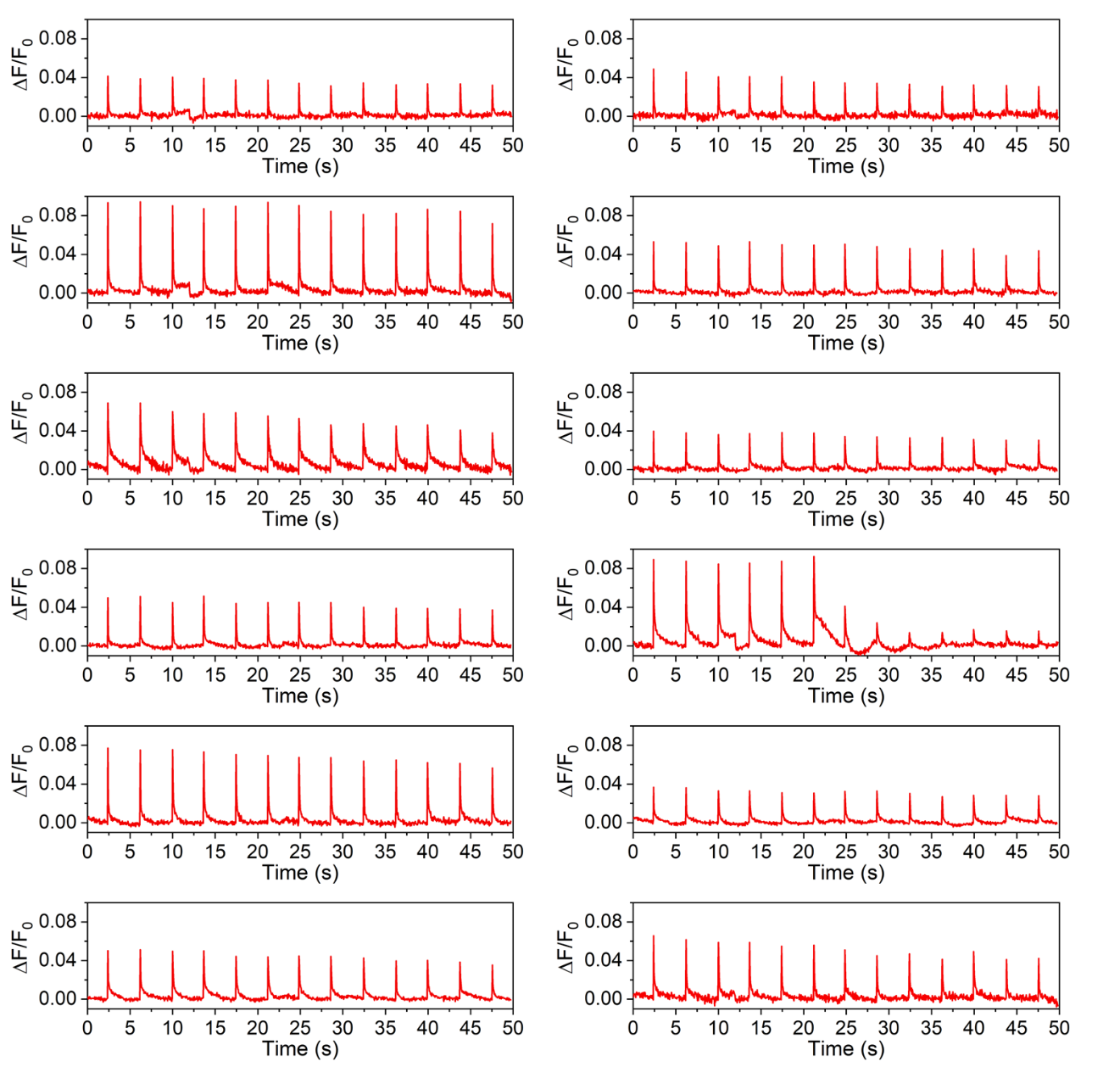


Figure. S8. Calcium traces obtained from 12 neurons under wide-field MIPA stimulation.


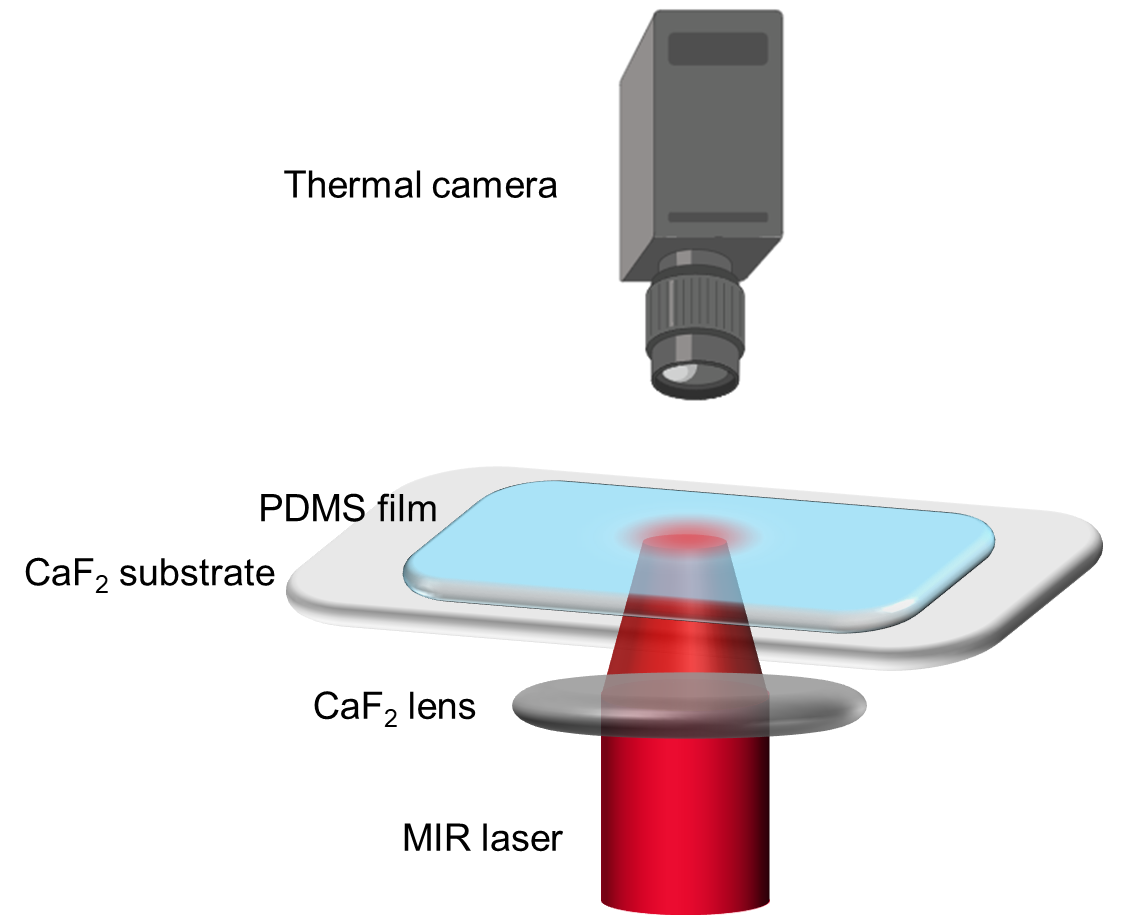


**Figure. S9**. Schematic of the temperature measurement by the thermal camera.

**Reference**

1. Li, J.; Lan, X.; Lei, S.; Ou-Yang, J.; Yang, X.; Zhu, B., Effects of carbon nanotube thermal conductivity on optoacoustic transducer performance. *Carbon* **2019,** *145*, 112-118.

2. Baac, H. W.; Ok, J. G.; Maxwell, A.; Lee, K. T.; Chen, Y. C.; Hart, A. J.; Xu, Z.; Yoon, E.; Guo, L. J., Carbon-nanotube optoacoustic lens for focused ultrasound generation and high-precision targeted therapy. *Sci Rep* **2012,** *2*, 989.
